# Small Cellular Particles from European Spruce Needle Homogenate

**DOI:** 10.3390/ijms24054349

**Published:** 2023-02-22

**Authors:** Marko Jeran, Anna Romolo, Vesna Spasovski, Matej Hočevar, Urban Novak, Roman Štukelj, Vid Šuštar, Matic Kisovec, Apolonija Bedina Zavec, Ksenija Kogej, Aleš Iglič, Polonca Trebše, Veronika Kralj-Iglič

**Affiliations:** 1 University of Ljubljana, Laboratory of Clinical Biophysics, Faculty of Health Sciences, SI-1000 Ljubljana, SloveniaVeronika Kralj-Iglič (V.K.I.); 2 University of Ljubljana, Laboratory of Physics, Faculty of Electrical Engineering, SI-1000 Ljubljana, Slovenia; 3 Institute of Molecular Genetics and Genetic Engineering, University of Belgrade, 11000 Belgrade, Serbia; 4 Institute for Metals and Technology, SI-1000 Ljubljana, Slovenia; 5 Theory Department, National Institute of Chemistry, SI-1000 Ljubljana, Slovenia; 6 Department of Molecular Biology and Nanobiotechnology, National Institute of Chemistry, SI-1000 Ljubljana, Slovenia; 7 University of Ljubljana, Faculty of Chemistry and Chemical Technology, SI-1000 Ljubljana, Slovenia; 8 University of Ljubljana, Laboratory of Clinical Biophysics, Faculty of Medicine, SI-1000 Ljubljana, Slovenia

**Keywords:** *Picea abies*, spruce needles, extracellular particles, extracellular vesicles, small cellular particles, interferometric light microscopy, exosomes, cell-to-cell communication, drug delivery

## Abstract

Small cellular particles (SCPs) are being considered for their role in cell-to-cell communication. We harvested and characterized SCPs from spruce needle homogenate. SCPs were isolated by differential ultracentrifugation. They were imaged by scanning electron microscope (SEM) and cryogenic transmission electron microscope (cryo TEM), assessed for their number density and hydrodynamic diameter by interferometric light microscopy (ILM) and flow cytometry (FCM), total phenolic content (TPC) by UV-vis spectroscopy, and terpene content by gas chromatography-mass spectrometry (GC-MS). The supernatant after ultracentrifugation at 50,000× *g* contained bilayer-enclosed vesicles whereas in the isolate we observed small particles of other types and only a few vesicles. The number density of cell-sized particles (CSPs) (larger than 2 μm) and meso-sized particles (MSPs) (cca 400 nm–2 µm) was about four orders of magnitude lower than the number density of SCPs (sized below 500 nm). The average hydrodynamic diameter of SCPs measured in 10,029 SCPs was 161 ± 133 nm. TCP decreased considerably due to 5-day aging. Volatile terpenoid content was found in the pellet after 300× *g*. The above results indicate that spruce needle homogenate is a source of vesicles to be explored for potential delivery use.

## 1. Introduction

Cell-to-cell communication is a fundamental process common to all biological kingdoms [1,2] and is necessary for the maintenance of homeostasis and the proper function of an individual organism. Cells continuously release small (sub-micron sized) particles (small cellular particles—SCPs) into the surroundings, recognize and uptake them from the surroundings and integrate the material into their substance which constitutes a cell-to-cell communication mechanism [3]. SCPs of different types (e.g., extracellular vesicles, protein aggregates, lipid droplets and viruses) can be present in the environment [4] and are suggested to be universal mediators of interaction between life domains [2,5]. SCPs reflect the content and metabolic state of the cells from which they originate [6,7,8,9]. Some SCPs show potential in delivery to target cells, enabling the transfer of specific cargo in a precise and controllable manner [10,11,12,13,14,15]. They can serve as vehicles for the transfer of biocompatible compounds, such as small interfering ribonucleic acid (siRNA) and antibodies, and also therapeutic agents including chemotherapeutic drugs, deoxyribonucleic acid (DNA) expression vectors, and others, through all kingdoms of life [1,2,6,10,14,15]. It was suggested that if proven to be of low toxicity, low immunogenicity and low allergenicity, they could be used in nanomedicine as drug delivery systems for the treatment of cancer and neurodegenerative diseases, such as Alzheimer’s disease, as well as inflammatory gut diseases, liver damage, hypoxic diseases, including myocardial infarction, renal ischemia-reperfusion and kidney injury [7,14,16,17,18].

To better understand the role of these processes in the functioning of cells and organisms, SCPs have been a subject of increasing interest in the last 30 years [19]. It was suggested that plant cells actively concentrate various molecules, including DNA, RNA, micro RNA, proteins, lipids and various metabolites in extracellular vesicles [20]. Membrane vesicles isolated from broccoli have been found to contain sulforaphane, which has cancer-protective properties [21]. Grapefruit SCPs contain naringenin, reported to possess antitumor properties [22], but also other metabolites, such as citric acid, glucose, sucrose myo-inositol, quinic acid, oxalic acid, glycolic acids, aucubin, as well as amino acids leucine and isoleucine [14]. Apple-derived nanoparticles contain flavonoids and furanocoumarins that are reported to exhibit anti-fungal and insecticide effects [23]. Exosome-like nanoparticles from strawberries were found to be rich in ascorbic acid which was shown to prevent oxidative stress in human mesenchymal stromal cells [24]. Curcuminoids have been detected in extracellular vesicles and nanovesicles isolated from Javanese ginger and turmeric which are known for their antioxidant properties [25]. It was found that extracellular vesicles are able to transport the cargo between plants and plant-invading pathogens, which is a mechanism of exosome-mediated immune response to the invading pathogen or parasite [6,26,27].

In this work, we considered European spruce (*Picea abies*) which has been acknowledged as a rich source of bioactive compounds traditionally used to treat various inflammatory disorders such as rheumatism, gout [28], skin ulcers and infected wounds [29]. Essential oils, which are distributed in different parts of the plant constitute monoterpenes, and about 70% are accounted to limonene, camphene, and pinene [30]. Antimicrobial activity of pinenes against Gram-positive bacteria, Gram-negative bacteria, and yeasts has been reported [31], as well as their antibiotic resistance modulator activity [32] and their anticoagulant, antitumor, antimicrobial, antimalarial, antioxidant, anti-inflammatory and anticancer properties [33,34,35]. The antimicrobial activity of limonene, which is mainly found in citrus fruit, has also been reported [36].

Membrane-enclosed nanoparticles have been found superior in drug delivery compared with other systems, with respect to site-targeting, sustained or controlled release and protection of cargo from clearance and degradation [37]. SCPs can be administered, transdermally, nasally and through the pulmonary route [37]. SCPs from natural sources may already contain the cargo and the membranes which simplify the harvesting process. It was of our interest to find and study SCPs from spruce needles as possible carriers of endogenous bioactive compounds. SCPs from *Picea abies* needles were to our best knowledge not yet systematically analyzed. Here, we have used the protocol developed for the isolation of extracellular vesicles by differential (ultra)centrifugation. We have found lipid bilayer-enclosed nanovesicles in the supernatant and in the isolate. We characterized the samples along the isolation process in terms of the morphology of particles, their size, number density, TPC and volatile terpenoid content.

## 2. Results

### 2.1. Visualization of the Samples

**Figure 1:**
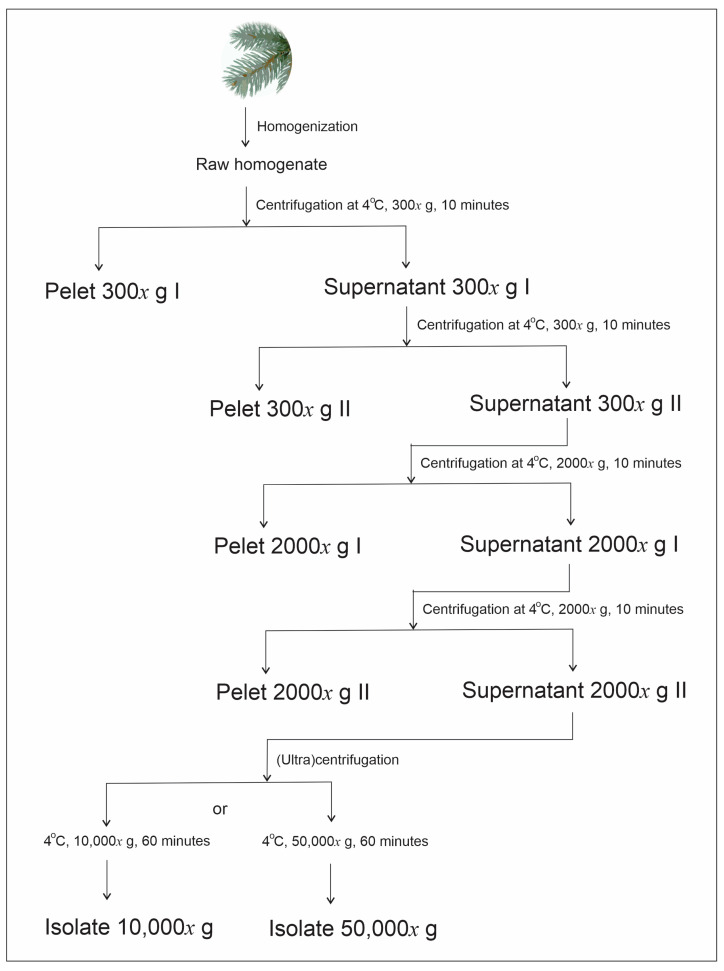
Scheme of the harvesting of SCPs. The centripetal acceleration of the centrifuge rotor is given in multiplicity of g (gravity acceleration near Earth’s surface).

Harvesting of SCPs from spruce needles is described in the Methods (Section 4.2) and schematically presented in Figure 1.

**Figure 2:**
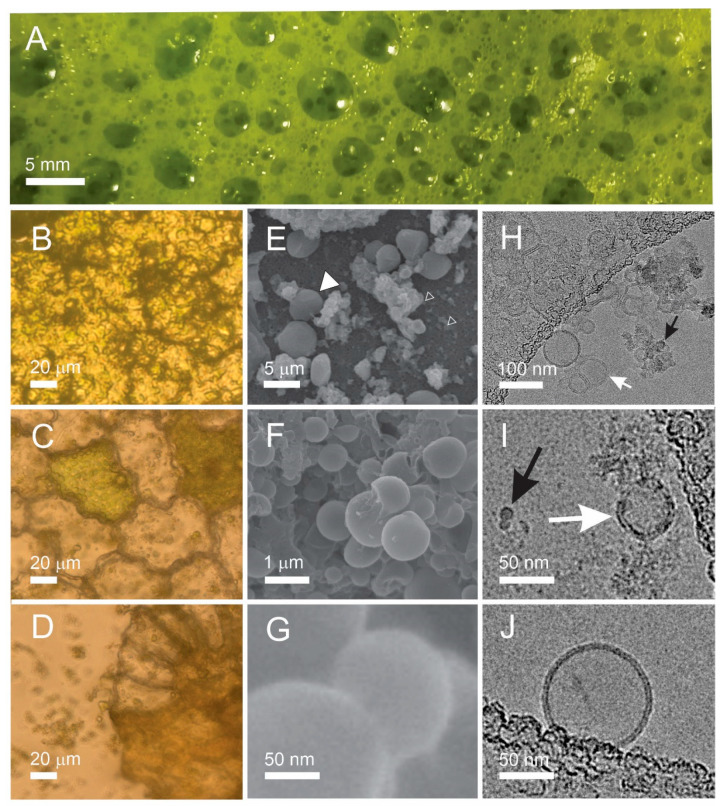
Micrographs of particles in samples undergoing differential (ultra)centrifugation. (**A**): foam after homogenization, (**B**): light microscope image of dried foam, (**C**,**D**): light microscope images of pellet after 300× *g* I, (**E**,**F**): SEM images of raw homogenate (from raw data SEM of spruce needle homogenate, https://doi.org/10.5281/zenodo.7437856, images 3 and 17, respectively), (**G**): SEM image of the isolate after 50,000× *g* (from raw data SEM of isolate from spruce needle homogenate, https://doi.org/10.5281/zenodo.7437856, image 9), (**H**) cryo-TEM image of supernatant from isolation of SCPs from spruce needle homogenate (pellet after 50,000× *g* ultracentrifugation) (from raw data Cryo-TEM of supernatant from isolation of SCPs from spruce needle homogenate, https://doi.org/10.5281/zenodo.7438603, Image S2/9), (**I**): isolate after 50,000× *g* (from raw data Cryo-TEM of isolate from spruce needle homogenate, https://doi.org/10.5281/zenodo.7438603, Image S9/4), (**J**): a single bilayer-enclosed nanovesicle in the supernatant after 50,000× *g* (from raw data Cryo-TEM of supernatant from isolation of SCPs from spruce needle homogenate, https://doi.org/10.5281/zenodo.7438603, Image S2/7). The full white triangle in Panel (**E**) points to particles with smooth contours, empty triangles in Panel (**E**) point to SCPs, white arrows in Panels (**H**,**I**) point to bilayer-enclosed vesicles and the black arrow in Panel (**I**) points to an electron-dense particle.

Figure 2 describes samples obtained along the processing of the needles. At homogenization, a foam was formed on top of the homogenate. It was gathered with a spoon and observed in the photographic image. Fresh foam showed millimeter-sized bubbles (Figure 2A) that were still present in an epruvette a day after the preparation. The absence of huge vesicles in the light microscope (LM) image of the fresh foam (Figure 2B) indicates that the bubbles have popped while being squeezed between the cover glasses. In the supernatant after 300× *g* I centrifugation of the raw homogenate, we observed numerous particles heterogeneous in size and shape including larger pieces of tissue showing cells (Figure 2C,D) filled with ~5 μm sized green particles with smooth contours (Figure 2C) and particles shed into the surrounding solution upon rupture of the cell walls (Figure 2D). In scanning electron microscope (SEM) images, particles with smooth contours were recognized in the raw homogenate (Figure 2E, full white triangle); however, smaller particles could also be observed (Figure 2E, empty white triangles). Raw homogenate (Figure 2F) and isolate after 50,000× *g* (Figure 2G) contained particles down to nanometer-sized ones mostly agglomerated into micrometer-sized lumps (Figure 2F). Cryogenic transmission electron microscope (cryo-TEM) images of the 50,000× *g* supernatant and isolate (Figure 2H–J) showed transparent bilayer membrane-enclosed vesicles (white arrows) but also electron-denser particles (Figure 2H,I, black arrows). The bilayer membrane could be resolved (Figure 2J). Amorphous material (Figure 2H,I) could derive from destructed tissue. Please see also the images of the raw data by cryo-TEM at https://doi.org/10.5281/zenodo.7438603, accessed on 18 February 2023, and by SEM at https://doi.org/10.5281/zenodo.7437855, accessed on 18 February 2023. The estimated proportion in numbers (vesicles/electron-dense particles) obtained by counting the particles in the cryo-TEM images of the supernatant was 9:1 (the numbers of vesicles varied between 0 and 80 per image and the numbers of electron-dense particles varied between 0 and 15), and in the isolate, it was 1:20 (the numbers of vesicles varied between 0 and 2 per image and the numbers of electron-dense particles varied between 10 and 30).

**Figure 3:**
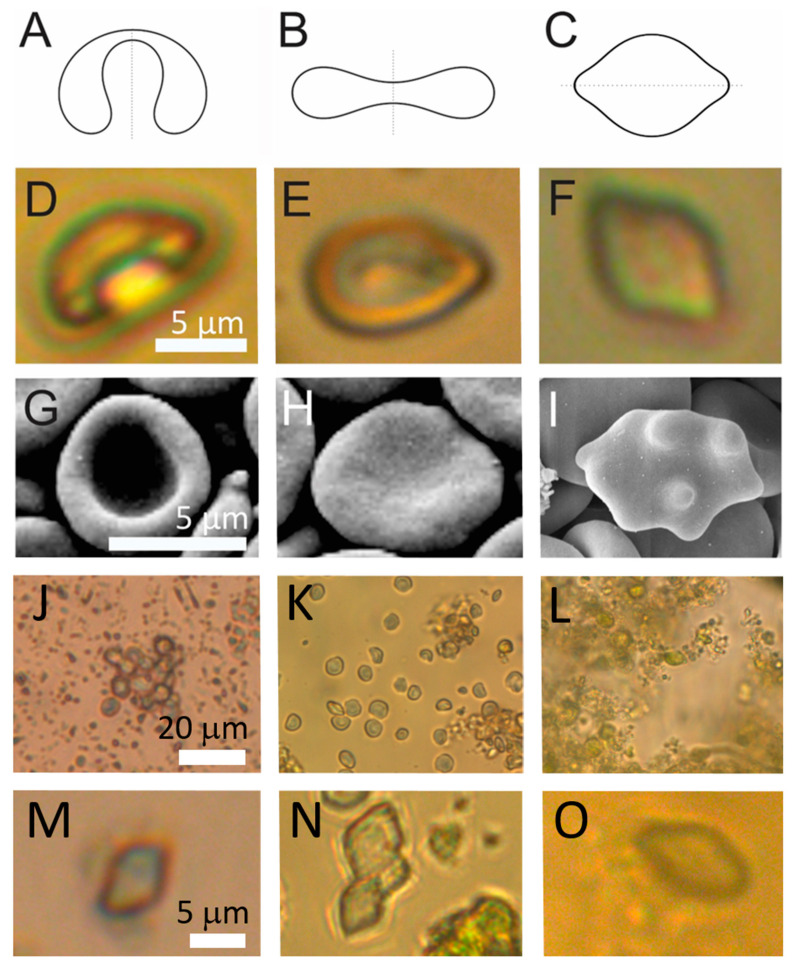
Examples of cell-sized particles (CSPs) with smooth contours in spruce needle homogenates. (**A**–**C**): theoretically calculated shapes that correspond to shapes with minimal membrane free energy (see [41,42]). (**D**–**F**,**J**): example CSPs from spruce homogenate, (**G**–**I**): example erythrocytes. (**J**–**L**): populations of CSPs from three independent samples and (**M**–**O**): examples of lemon-shaped CSPs from these samples. The parameters for calculated shapes were A: *v* = 0.6, <*h*> = 0.650, B: *v* = 0.6, <*h*> = 1.040 (from Kralj-Iglič et al. [41]), C: *v* = 0.95, <*h*> = 1.0375.

Shapes of particles in which a fluid interior is enclosed within a membrane are theoretically determined by the minimization of the membrane free energy [38,39,40]. Shapes with minimal membrane free energy (Figure 3A–C) can be characterized by the volume-to-surface area proportion represented by the relative volume *v*, and the average mean curvature <*h*> [41,42]. Shapes of some of the particles in spruce homogenate had smooth contours (Figure 3D–F) corresponding to theoretically calculated shapes. Examples of these particles were found in raw homogenate and in pellets after 300× *g* I and 2000× *g* I but not in the pellet after 50,000× *g*.

The parameter <*h*> in Figure 2 increases from left to right as given in the figure caption. Stomatocytic and discocytic shapes (Figure 3A and Figure 3B, respectively) can be found in both, spruce needle homogenate (Figure 3D and Figure 3E, respectively) and in erythrocyte suspension (Figure 3G and Figure 3H, respectively), while at higher average mean curvature the lemon shape (Figure 3C) found in spruce homogenate (Figure 3F) differed from the echinocytic shape of the erythrocyte (Figure 3I).

As regards the abundance of these shapes, stomatocytic and discocytic shapes were more abundant. In the viewframes with many particles (Figure 3J–L) we can see about 15 such particles in Panel J, about 20 such particles in Panel K and about 10 such particles in Panel L while numerous smaller particles can be observed (more than 100 in each of these frames). The lemon-shaped particles were even less abundant than the stomatocytic and discocytic particles as none of them could be outlined in these frames (Figure 3J–L). However, on close inspection, examples of such shapes could be found (Figure 3M–O).

### 2.2. Determination of Number Density and Size of Particles

**Figure 4:**
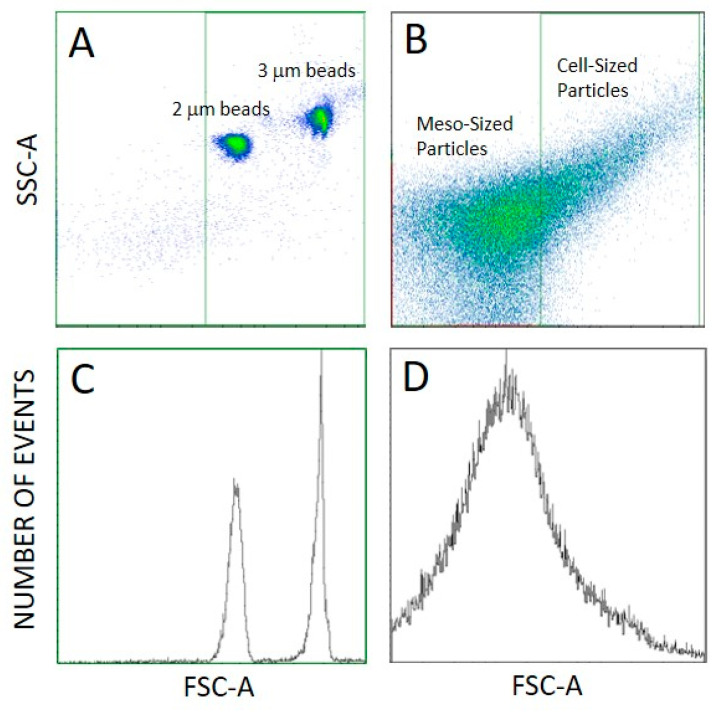
Setting of the gates in FCM. (**A**): Scatter plot of 2 and 3 µm sized MACSQuant Calibration Beads. (**B**): Scatter plot of a sample, (**C**,**D**): respective distribution representations.

We distinguished three types of detected particles as regards the size: cell-sized particles (CSPs) (larger than about 2 µm), meso-sized particles (MSPs—particles at the lower limit of detection of FCM to about 2 µm), corresponding to the gates set in flow cytometry (Figure 4B,D), and SCPs (smaller than about 500 nm) that can be detected by interference light microscope (ILM). The majority of particles in the samples were the smallest ones (SCPs) as their number density was four orders of magnitude higher than the number densities of MSPs and CSPs (Table 1). Furthermore, the number density of MSPs was larger than the number density of CSPs (Table 1). We observed no trend in the number densities of SCPs, MSPs and CSPs nor in the hydrodynamic diameter D_h_ of SCPs along the isolation process (Table 1).

Measurements were made on three independent samples, with each sample in triplicate. Average values and standard deviations for the number densities were calculated for the three measurements of the same sample. The standard deviation for D_h_ was taken as the largest from the triplicate. For FCM the samples were diluted 10× and for ILM the samples were diluted 30×. The number densities reported were corrected for respective dilution factors.

### 2.3. Analysis of the Content of the Samples

#### 2.3.1. Determination of the Ultraviolet-Visual Absorption

Table 2 shows ultraviolet-visual absorption in the samples subjected to a differential centrifugation process. After 5 days, the content of compounds that absorb light at 760 nm was considerably lower than in fresh samples (Table 2).

The measurements were performed on three independent samples, each measurement was performed in triplicate. UV-vis: ultraviolet-visual.

#### 2.3.2. Determination of Volatile Terpenoid Content by Gas Chromatography-Mass Spectrometry Analysis

We analyzed the presence of terpenes along the isolation process in three independent samples. We found notable signals only in the pellet after 300× *g* I centrifugation (Table 3).

## 3. Discussion

We isolated SCPs from a homogenate of spruce needles, observed them with different microscopic techniques and analyzed some of their properties (number density, size, TPC and volatile terpenoid content) during the isolation process by differential ultracentrifugation. We imaged two independent samples (starting from needles from the same tree at different times) by cryo-TEM. In our first attempt, we did not observe any bilayer-enclosed vesicles in the isolate or in the supernatant. In our second attempt, we observed small round double-layer-enclosed vesicles with diameters of about 100 nm in supernatant and in isolate (Figure 2H–J). We concluded that it is possible to obtain membrane-enclosed vesicles in the samples (as they were actually observed) but these vesicles were scarce and were not readily obtained in sufficient quantity to be observed by cryo-TEM every time we made the samples. SEM images were made on three independent samples: two isolates and one homogenate. The raw data images on the isolate https://doi.org/10.5281/zenodo.7437856, accessed 18 February 2023, # 1–19 pertain to one isolate and # 20–36 pertain to the other isolate. It can be seen from these images that the particles were heterogeneous in size and that they lumped together in SEM images. Yet, the particles in the range of 50–300 nm were present in the samples which is in agreement with the average hydrodynamic diameter measured by a batch method ILM (Table 1). The supernatant after ultracentrifugation contained a larger number of vesicles while the isolate (pellet) contained small particles of other types but only a few vesicles (Figure 2, raw data). The number density of CSPs and MSPs was more than three orders of magnitude lower than the number density of SCPs (Table 1) and the number density of CSPs was smaller than the number density of MSPs (Table 1).

Starting from three independent samples from the same tree, we observed no trend in the number density of CSPs, MSPs or SCPs along the isolation process (Table 1), or in phenolic content along the isolation process (Table 2). Volatile terpenoid content was notable only in the pellet after 300 g.

Phenolic acids are studied for their potential antioxidant and anti-inflammatory effects while terpenes are studied for their potential analgesic, anticonvulsant and anti-inflammatory effects [43]. Our results suggest that we lose terpenes along the isolation procedure. We have applied a centrifugation protocol that is commonly used for the isolation of extracellular vesicles deriving from different samples [44]. However, the isolation procedure affects the identity of the particles in the isolate, so higher yields of the SCPs and lower losses of terpenes would be expected if the centrifugation protocols were optimized.

The observed vesicles were membrane-bound and seemed transparent in cryo-TEM images (Figure 1). It is possible that they were formed during the processing, e.g., by capturing the liquid with dissolved molecules. Since we observed that the vesicles were more numerous in the supernatant than in the isolate, they could have been moving up or too slowly moving down during the centrifugation or they could have been destroyed in the isolate by high centrifugal force pressing them against the bottom of the tube. ILM showed that the isolates contained the highest number density of SCPs (Table 1), most likely of non-vesicular form. Further justification and experiments are needed to validate these possibilities.

Figure 3 shows that CSPs observed by LM attained shapes that are characteristic of membrane-enclosed entities without internal structure. In such vesicles, the shape is determined by the minimum of the membrane elastic energy [41,42]. We found reports on smooth shapes of particles with similar sizes and shapes attributed to starch granules [45]. Furthermore, erythrocytes are particles with structureless interiors in which the shape is determined by the minimum of the membrane free energy (Figure 3). The stomatocyte (Figure 2D)–discocyte (Figure 3E)–echinocyte (Figure 3F) transformation can be driven by the change of the relative volume and average mean curvature of the membrane <*h*>. The stability of the stomatocyte and the discocyte shapes can be described by the spontaneous curvature or difference between the outer and the inner membrane layer areas but not of the echinocyte shape. The echinocyte shape can be explained by including the shear energy of the membrane skeleton [46]. The shape of the particles observed in our samples had two protrusions on the poles of the particle (Figure 3F) which however correspond to the minimum of the elastic energy within the branch of the shapes symmetric with respect to the equatorial plane. The stomatocyte–discocyte sequence has been observed also in giant phospholipid vesicles, similar to the erythrocytes and particles observed in this work, for increasing <*h*>. In giant phospholipid vesicles, further increasing <*h*> yields pear shapes that are asymmetric with respect to the equatorial plane. After reaching the global energy minimum, the symmetric and asymmetric branches separate. Lowering the energy of the symmetric branch can be a consequence of the lateral redistribution of membrane constituents or of the spontaneous curvature of the membrane that shifts the symmetric branch below the asymmetric one.

In many reports, SCPs were divided into three types (apoptotic bodies—fragments of the decaying cell; microvesicles originating from the budding of the plasma membrane and exosomes originating from internal compartments, e.g., endosomes) [19]. Microvesicles and exosomes were distinguished by their size (100 nm and more for microvesicles, and 50–150 nm for exosomes). A more recent division of extracellular vesicles into small and large [47] connects the size to the origin indicating that the plasma membrane blebbing leads to large extracellular vesicles larger than 100 nm while small extracellular vesicles are exosomes deriving from internal compartments). However, there is an overlaying interval between 100 and 150 nm which pertains to both types. It seems reasonable that particles in the range of micrometer size are not likely to derive from internal compartments because the internal compartments are of that order of size. However, microvesicles smaller than 100 nm have been observed in erythrocytes (see [41] and included references). Extracellular vesicle isolates are in general heterogeneous in size and composition. Within the available characterization and methods used in this work, we cannot distinguish microvesicles from exosomes–or–small extracellular vesicles from large ones. Furthermore, we do not know how many of the particles detected by the batch methods are membrane-enclosed vesicles and not some other types of particles (such as lipoproteins or granules). Therefore we referred to them as SCPs.

The method used to determine phenols can be biased since the presence of proteins or other components as nitrogen-containing compounds can not be ruled out.

## 4. Materials and Methods

### 4.1. Preparation of Homogenates

#### Preparation of Homogenate from Spruce Needles for Isolation of Small Cellular Particles

A sample of 50 g of spruce branches was immersed for 1 h in 200 g of water with 0.2 g sodium hypochlorite (NaClO) (at about 30 °C and pH 6.5). The branches were then thoroughly washed with water (pH = 6). The needles were cut off from the branches. Then, 300 mL of ultraclean water (B Braun, Meisungen, Germany) was added to the needles and homogenized in a stirrer Bullet Blender (KOIOS 850W Smoothie, Homeland Housewares, Los Angeles, CA, USA) for 30 s. To remove larger particles, the homogenate was filtered through 0.5 mm nylon net cloth.

### 4.2. Isolation of Small Cellular Particles

SCPs were isolated by differential centrifugation as adapted from the protocol for the isolation of extracellular vesicles (EVs) from [44]. The filtered homogenate was centrifuged twice at 300× *g* and 4 °C for 10 min in the centrifuge Centric 260R with rotor RA 6/50 (Domel, Železniki, Slovenia) by using 50 mL conical centrifuge tubes (ref. S.078.02.008.050, Isolab Laborgeräte GmbH, Germany). The supernatant of the second centrifugation was centrifuged twice at 2000× *g* and 4 °C for 10 min in the centrifuge Centric 400R with rotor RS4/100 (Domel, Železniki, Slovenia), using 15 mL conical centrifuge tubes (ref. S.078.02.001.050, Isolab Laborgeräte GmbH, Eschau, Germany). Then, the supernatant was centrifuged at 10,000× *g* or 50,000× *g* and 4 °C for 60 min in Beckman L8-70M ultracentrifuge, rotor SW55Ti (Beckman Coulter, Brea, CA, USA) using thin-wall polypropylene centrifuge tubes (ref. 326819, Beckman Coulter, Brea, CA, USA). The respective isolates were obtained by dissolving pellets in a small amount of solvent (e.g., 80 µL).

### 4.3. Visualization of Samples

Since the particles in the samples were heterogeneous in size, we visualized them by different microscopic techniques. We used light microscopy (LM) to visualize cell-sized particles (CSPs), scanning electron microscopy (SEM) to visualize particles ranging from cell-sized to nano-sized particles, and cryogenic transmission electron microscopy (cryo-TEM) to visualize SCPs.

#### 4.3.1. Light Microscopy

Homogenates were imaged with Nikon EM CCD inverted light microscope (Eclipse TE2000-S, Tokyo, Japan) with a digital camera system: spot boost (Visitron Systems, Pucheim, Germany).

#### 4.3.2. Scanning Electron Microscopy

The samples were fixed with OsO_4_ as adapted from [48]. Samples were placed on 0.05-micron mixed-cellulose-esters’ filters (Sterlitech, Auburn, AL, USA) and incubated in 39.3 mM double distilled water solution of OsO_4_ for 2 h. Then they were washed 3 times with distilled water (10 min each), dehydrated in graded series of ethanol (30%, 50%, 70%, 80%, 90%) and absolute ethanol, each step 10 min. Absolute ethanol was replaced twice. Then they were washed in hexamethyldisilazane (mixed with absolute ethanol; 30% and 50%) and in absolute hexamethyldisilazane, each step 10 min. The samples were left to dry in air overnight. For examination under JSM-6500F Field Emission Scanning Electron Microscope (JEOL Ltd., Tokyo, Japan), the samples were sputtered with Au/Pd (PECS Gatan 682). Erythrocytes in Figure 2 were imaged as described in [49].

#### 4.3.3. Cryogenic Transmission Electron Microscopy

C-flat™ 2/2, 200 mesh holey carbon grids (Protochips, Morrisville, NC, USA) were glow discharged: 20 mA, 60 s, positive polarity, air atmosphere (GloQube^®^ Plus, Quor-um, Laughton, UK). Then, 3 µL of the sample was applied to the grid, blotted, and vitrified in liquid ethane on Vitrobot Mark IV (Thermo Fisher Scientific, Waltham, MA, USA). Vitrobot conditions were set to 100% relative humidity, 4 °C, blot force: 2 and blot time: 7 s. Samples were visualized under cryogenic conditions using a 200 kV Glacios microscope with a Fal-con 3EC detector (Thermo Fisher Scientific, Waltham, MA, USA).

### 4.4. Determination of Amount and Size of Particles

Since the particles in all the samples measured (spruce needle homogenate and centrifugation pellets and supernatants) were heterogeneous in size, we distinguished three populations subjected to different assessment methods. We used flow cytometry (FCM) to assess CSPs and middle-sized particles in the range between 400 nm and 1 µm (MSPs), and interferometric light microscopy (ILM) to assess SCPs in the size range of 70–500 nm.

#### 4.4.1. Flow Cytometry

FCM measurements were performed as adapted from the protocol for microalgae [50] by using a MACS QUANT flow cytometer (Miltenyi, Bergisch-Gladbach, Germany). Standard beads of size 2 and 3 µm (MACSQuant Calibration Beads, ref: 130-093-607) were imaged to help in setting the gates (Figure 4A,C). Two gates were set distinguishing between CSPs (larger than about 2 µm) and MSPs (400 nm to about 2 µm) (Figure 4B). Lasers were set at 458 V (FSC), 467 V (SSC), the main trigger was set at SSC 1.800 and the secondary trigger was set at FSC 3.0.

#### 4.4.2. Interference Light Microscopy

**Figure 5:**
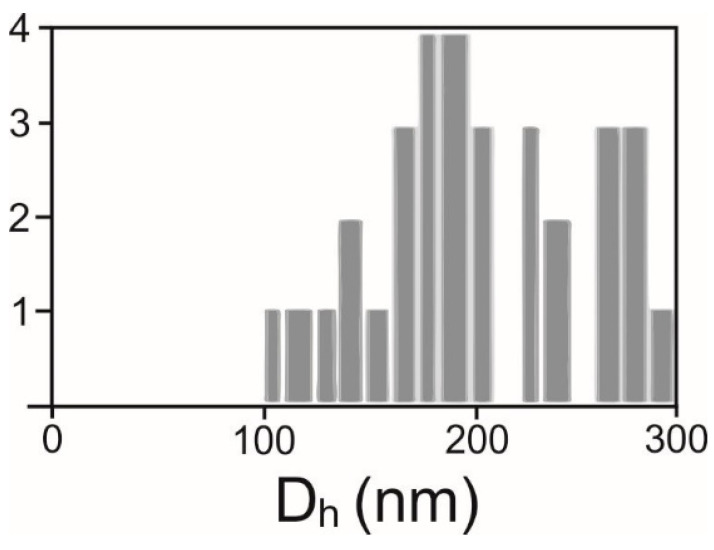
Distribution of hydrodynamic diameters D_h_ in an example sample.

The average hydrodynamic diameter (D_h_) and the number density of SCPs were determined by interferometric light microscopy (ILM) using Videodrop (Myriade, Paris, France) as described in [51]. The signal of the medium (ultraclean water) was under the detection limit. We used the particle detection threshold value 3.5. Before measurement, the samples were filtered sequentially through 800, 450 and 200 nm filters. Then, a 7 μL drop of sample (supernatant or isolate from the isolation of SCPs) was placed between cover glasses and illuminated by a 2W blue light emitting diode light. The scattered light interfered with the incoming light and the interference pattern was detected by a complementary metal-oxide–semiconductor high-resolution high-speed camera. The incident light signal was subtracted. In the obtained pattern, the contrasting black and white spots were recognized as a particle. The number of particles in a given volume (typically 15 pL) was detected as the number density of the particles. Particles were tracked by video. The particle trajectories were analyzed. It was taken that particles undergo Brownian motion depending on the temperature of the sample and on the diffusion coefficient (D) of the particle. D is taken to be proportional to the mean square displacement (d) of the particle between two consecutive frames taken in the time interval ∆t, <d^2^(∆t)> = <4D ∆t>. The hydrodynamic diameter was estimated by using the Stokes-Einstein relation D_h_ = kT/3πηD assuming that the particles were spherical. Each particle that was included in the analysis was tracked and processed individually. Statistical analysis yielded the average hydrodynamic diameter and other statistical parameters (e.g., standard deviation and the number of particles tracked) (Figure 5). The processing of the images and of the movies was performed by the associated software QVIR 2.6.0 (Myriade, Paris, France).

### 4.5. Analysis of the Total Phenolic Content of the Samples

#### 4.5.1. Determination of Absorbance of Light at 760 nm

The content of phenolic compounds in samples was determined by following the procedures reported in [52]. Aliquots of the samples (2.5 µL) were mixed with 10-times diluted Folin–Ciocalteu reagent (2 N, Sigma Aldrich, St. Louis, MO, USA) (12.5 µL) and 10 µL of 7.5% aqueous solution of sodium carbonate (prepared from ACS reagent ≥99.5 %, Sigma-Aldrich, Taufkirchen, Germany). The mixture was allowed to stand for 30 min in a dark place at room temperature. Then the absorbance at 760 nm was assessed spectrophotometrically with Nanodrop One C (Thermo Scientific, Waltham, MA, USA). Ultraclean water was used as a blank. The calibration curve was made from standard gallic acid solutions at concentrations 2, 10, 18, 26, 34, 42, 58, 66, 82 and 90 µg/mL. The results were expressed as quantity of gallic acid per quantity of plant weight. The method has previously been used to determine TPC in natural samples [53]; however, there could also be a contribution of the presence of proteins or other components that contain nitrogen.

#### 4.5.2. Determination of Volatile Terpenoid Content by Gas Chromatography-Mass Spectrometry

Water-based aliquots (up to 0.5 mL) with biologically active compounds were dried for one hour at 40 °C with miVac modular concentrator (SP Scientific, Warminster, PA, USA). Pellets were resuspended in methanol (0.5 mL per each sample). To assess the composition of the dry phase, the modified method for the identification of terpenoids based on gas chromatography with mass spectrometry (GC-MS) was applied by using gas chromatograph 6890 (GC; Agilent Technologies Inc., Santa Clara, CA, USA) with a series of quadrupole mass spectrometers 5973 Network (MS; Agilent Technologies Inc., Santa Clara, California, USA) in electronic ionization mode at conditions: capillary column Rxi-35 Sil MS (Restek, Center County, PA, USA), splitless GC mode, oven temperature 50 °C (2 min hold) to 250 °C at 9 °C × min-1, carrier gas: helium (Messer, Bad Soden, Germany) with flow rate 1.2 mL × min^−1^, mass scan from *m*/*z* 50 to *m*/*z* 550 and ion source; 230 °C total ion count. The active substances were identified by comparison with standards and retention times and by comparison with mass spectra from computer libraries (HPCH2205, Wiley7N and FENSC3) [54]. The values were calculated on the basis of the calibration curves of the terpenoid standards #1 and #2, which are a mixture of 19 and 2 terpenoids, respectively. Relative retention times t_R_ (in minutes) of terpenes from GC-MS with respect to standard solutions (Restek standard #1 and #2*) were α-Pinene (2.197), Camphene (3.659), (-)-β-Pinene (4.177), β-Myrcene (4.380), δ-3-Carene (4.654), α-Terpinene (4.888), D-Limonene (5.083), Eucalyptol (1,8-Cineole)* (5.189), Ocimene (5.342), γ-Terpinene (5.713), Terpinolene (6.185), Linalool (6.592), (-)-Isopulegol (7.629), Geraniol (9.318), β-Karyophyllene (11.275), α-Humulene (11.886), Nerolidol (13.129), (-)-Caryophyllene oxide* (14.110), and (-)-α-Bisabolol (15.414).

## 5. Conclusions

We found that spruce needles contain material that can spontaneously form fragile nanovesicles enclosed by a bilayer membrane. Since the fluid formed during the homogenization of the needles is rich in molecules that are considered beneficial to health, optimization of sample processing is a prerequisite to avoid loss of the cargo and increase the vesicle yield. 

## Figures and Tables

**Table 1:** The number density of SCPs, MSPs and CSPs and hydrodynamic diameter D_h_ of SCPs in fresh samples as measured by ILM and FCM.

	SCPs			MSPs	CSPs
Sample	Number Density (/mL)	D_h_ (nm)	Number of Particles Tracked	Number Density (/mL)	Number Density (/mL)
Supernatant 300× *g* I	(7.82 ± 1.85) × 10^10^	162 ± 114	2892	(11.0 ± 2.74) × 10^6^	(3.20 ± 0.45) × 10^6^
Supernatant 2000× *g* I	(6.86 ± 1.42) × 10^10^	159 ± 134	2413	(10.1 ± 5.28) × 10^6^	(0.77 ± 0.33) × 10^6^
Isolate 10,000× *g*	(11.9 ± 2.03) × 10^10^	172 ± 101	2913	(10.7 ± 5.39) × 10^6^	(2.12 ± 0.75) × 10^6^
Isolate 50,000× *g*	(3.64 ± 0.61) × 10^10^	152 ± 107	1811	(11.4 ± 7.45) × 10^6^	(1.68 ± 0.70) × 10^6^

**Table 2:** Ultraviolet-visual light absorbance in the samples subjected to differential centrifugation process.

Sample	UV-Vis Light Absorbance (mg Gallic Acid/ g Spruce Needles) of Fresh Samples	UV-Vis Light Absorbance (mg Gallic Acid/ g Spruce Needles) of Samples Aged 5 Days
Supernatant 300× *g* I	38.47 ± 1.73	23.50 ± 3.22
Supernatant 2000× *g* I	41.78 ± 3.42	19.55 ± 6.25
Isolate 10,000× *g*	45.50 ± 1.36	20.93 ± 5.21
Isolate 50,000× *g*	42.60 ± 5.22	20.38 ± 1.68

**Table 3:** Content of volatile compounds in pellet 300× *g* I represented by peak areas.

		Pellet 300× *g* I 1	Pellet 300× *g* I 2	Pellet 300× *g* I 3	Average ± SD
Compound	t_R_ (minutes)	Peak area (mV/s)			
α-Pinene	2.30	3,553,050	4,463,328	3,964,389	3,993,589 ± 455,841
Camphene	3.46				
(-)-β-Pinene	4.07	975,290	2,401,657	1,911,749	1,762,899 ± 724,740
β-Myrcene	4.29				
3-Carene	4.56				
α-Terpinene	4.89				
D-Limonene	5.00	3,759,264	528,512	4,610,539	2,966,105 ± 2,153,500
Eucalyptol	5.27	806,207	256,749	1,102,675	721,877 ± 429,222
p-cymene	5.35				
o-Cymene	5.29				
γ-Terpinene	5.63				
Terpinolen	6.10				
Linalool	6.52				
(-)-Isopulegol	7.53				
Geraniol	9.32				
β-Caryophyllene	11.18	618,754	4,965,629	7,195,659	4,260,014 ± 3,344,748
α-Humulene	11.79				
Nerolidol	13.37				
Caryophyllene oxide	14.1				
Guaiol	14.1				
α-Bisabolol	15.26				

t_R_: retention time. The measurements were performed on three independent samples (pellet 300× *g* I 1, pellet 300× *g* I 2, and pellet 300× *g* I 3).

## Data Availability

Raw data on cryo-TEM are available at https://doi.org/10.5281/zenodo.7438603 accessed on 18 February 2023. Raw data on SEM are available at https://doi.org/10.5281/zenodo.7437855 accessed on 18 February 2023.
